# 1α,6β,7β,11α,15β-Penta­hydr­oxy-7α,20-ep­oxy-*ent*-kaur-16-ene

**DOI:** 10.1107/S1600536810000619

**Published:** 2010-01-13

**Authors:** Chuang Feng, Lan-Qing Guo, Fu-Lin Yan, Jian-Min Cui, Xue-Mei Di

**Affiliations:** a School of Pharmacy, Xinxiang Medical University, Xinxiang, Henan 453003, People’s Republic of China; b School of Nursing, Xinxiang Medical University, Xinxiang, Henan 453003, People’s Republic of China; c Henan College of Traditional Chinese Medicine, Zhengzhou, Henan 450008, People’s Republic of China

## Abstract

The title compound, C_20_H_30_O_6_, a natural *ent*-kaurane diterpenoid, named nervosanin B, was obtained from the medicinal plant *Isodon serra*. It is composed of four rings with the expected *trans* and *cis* junctions. One of the six-membered rings is in a chair conformation, the other two are in boat conformations and the five-membered ring adopts an evenlope conformation. The mol­ecules stack along the *a* axis and are linked together by inter­molecular O—H⋯O hydrogen bonds. Two intramolecular O—H⋯O interactions also occur.

## Related literature

For related literature on genus *Isodon* and diterpenoids, see: Sun *et al.* (2001 ▶); Wang *et al.* (1994 ▶); Yan *et al.* (2008 ▶). For bond-length data, see: Allen *et al.* (1987 ▶).
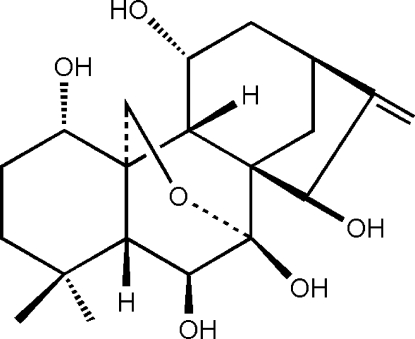


## Experimental

### 

#### Crystal data

C_20_H_30_O_6_
*M* *_r_* = 366.44
Monoclinic, 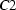

*a* = 21.581 (11) Å
*b* = 6.111 (3) Å
*c* = 14.080 (7) Å
β = 99.129 (8)°
*V* = 1833.3 (16) Å^3^
*Z* = 4
Mo *K*α radiation
μ = 0.10 mm^−1^
*T* = 93 K
0.60 × 0.18 × 0.14 mm

#### Data collection

Rigaku AFC10/Saturn724+ diffractometer
Absorption correction: multi-scan (*ABSCOR*; Higashi, 1995 ▶) *T*_min_ = 0.944, *T*_max_ = 0.987
7255 measured reflections
2291 independent reflections
1853 reflections with *I* > 2σ(*I*)
*R*_int_ = 0.052

#### Refinement

*R*[*F* ^2^ > 2σ(*F* ^2^)] = 0.039
*wR*(*F* ^2^) = 0.079
*S* = 1.00
2291 reflections
257 parameters
1 restraint
H atoms treated by a mixture of independent and constrained refinement
Δρ_max_ = 0.31 e Å^−3^
Δρ_min_ = −0.21 e Å^−3^

### 

Data collection: *CrystalClear* (Rigaku, 2008 ▶); cell refinement: *CrystalClear*; data reduction: *CrystalClear*; program(s) used to solve structure: *SHELXS97* (Sheldrick, 2008 ▶); program(s) used to refine structure: *SHELXL97* (Sheldrick, 2008 ▶); molecular graphics: *SHELXTL* (Siemens, 1995 ▶); software used to prepare material for publication: *SHELXTL*.

## Supplementary Material

Crystal structure: contains datablocks global, I. DOI: 10.1107/S1600536810000619/hg2628sup1.cif

Structure factors: contains datablocks I. DOI: 10.1107/S1600536810000619/hg2628Isup2.hkl

Additional supplementary materials:  crystallographic information; 3D view; checkCIF report

## Figures and Tables

**Table 1:** Hydrogen-bond geometry (Å, °)

*D*—H⋯*A*	*D*—H	H⋯*A*	*D*⋯*A*	*D*—H⋯*A*
O2—H2O⋯O5^i^	0.93 (3)	1.74 (3)	2.655 (3)	167 (3)
O4—H4O⋯O6^ii^	0.87 (3)	2.02 (3)	2.696 (3)	133 (2)
O3—H3O⋯O6^iii^	0.89 (3)	1.92 (3)	2.787 (3)	164 (3)
O5—H5O⋯O2	0.89 (3)	1.80 (3)	2.652 (3)	160 (3)
O6—H6O⋯O3	0.78 (3)	1.93 (3)	2.674 (3)	157 (3)

Symmetry codes: (i) 
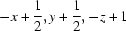
; (ii) 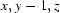; (iii) 
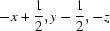
.

**Fig. 1.**
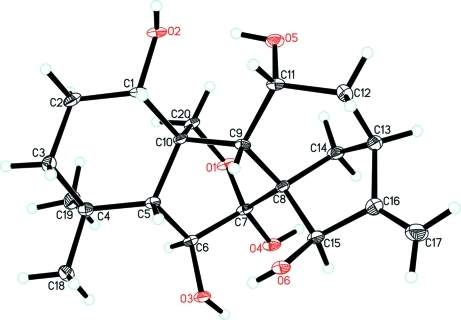
A view of the molecular structure of compound. Displacement ellipsoids are drawn at the 50% probability level.

**Fig. 2.**
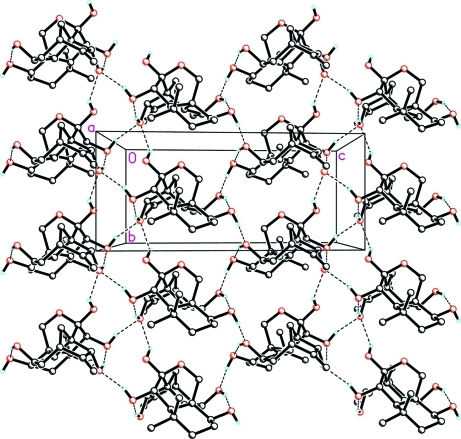
The crystal packing of compound, viewed along the a axis, showing the O—H···O hydrogen bonds as dashed lines.

## supplementary crystallographic information

### Comment

The title compound, 1α,6β,7β,11α,15β-Pentahydroxy-7α,20-epoxy- *ent*-kaur-16-ene is a natural *ent*-kaurane diterpenoid. It has been reported previously from *Isodon nervosa* (Wang *et al.*, 1994; Yan *et al.*, 2008) and its structure was postulated from spectroscopic methods (Wang *et al.*, 1994). Recently, it was also isolated from the medicinal plant *Isodon serra*, and its crystal structure analysis has been undertaken. The molecular structure is presented in Fig. 1. The molecule contains three six-membered rings (*A*,*B* and *C*) and a five-membered ring (*D*). There is a *trans* junction between ring *A* (C1—C5/C10) and ring *B* (C5—C10); *cis* junctions are present between ring *B* and ring *C* (C8/C9/C11—C14), and ring *C* and ring *D* (C8/C13—C16). Ring *A* adopts chair conformation, with an average torsion angles of 50.6 (3) °. Rings *B* and *C* adopt boat conformations because of the formation of the oxygen bridge at C-7 and C-20. Ring *D* shows an envelope conformation. In addition, the six-membered rings O1/C20/C10/C5—C7 and O1/C7—C10/C20 both adopt boat conformations.

The bond lengths are within expected ranges (Allen *et al.*, 1987), with averages values (Å): C*sp*^3^—C*sp*^3^ = 1.542 (3), C*sp*^3^—C*sp*^2^ = 1.521 (4), C*sp*^2^—C*sp*^2^ (CC) = 1.312 (4), C*sp*^3^—O = 1.435 (3). Compound contains ten chiral centers at C1(*S*), C5(*R*), C6(*S*), C7(*S*), C8(*S*), C9(*S*), C10(*S*), C11(*R*) C13(*S*) and C15(*R*). Although the absolute configuration could not be reliably determined from anomalous dispersion effects, the negative optical rotation showed this compound to be in the *ent*-kaurane series as reported in genus *Isodon* (Sun *et al.*, 2001), rather than in the kaurane series, and so allowed us to assign the correct configuration. In the crystal structure, the molecular packing is stabilized by O2—H···O5, O4—H···O6, O3—H···O6, O5—H···O2 and O6—H···O3 hydrogen bonds along the *a* axis and are linked by O—H···O hydrogen bonds (Table 1 and Fig. 2).

### Experimental

The dried and crushed leaves of *Isodon serra* (Maxim.) (10 kg, collected from Tongbai Prefecture, Henan Province, China) were extracted four times with Me_2_CO/H_2_O (7:3, *v*/*v*) at room temperature over a period of six days. The extract was filtered and the solvent was removed under reduced pressure. The residue was then partitioned between water and AcOEt. After removal of the solvent, the AcOEt residue was separated by repeated silica gel (200–300 mesh) column chromatography and recrystallization from CHCl_3_/CH_3_OH (10:1), giving 45 mg of compound (m.p. 531–533 K. Optical rotation: [α]_D_^20^ -50.6 ° (c 0.15, CH_3_OH). Crystals suitable for X-ray analysis were obtained by slow evaporation of a solution of the compound in CH_3_OH at room temperature.

### Refinement

All H atoms were included in calculated positions and refined as riding atoms, with C—H = 0.98Å (CH_3_), 0.99Å (CH_2_), 0.95Å (═CH_2_), 1.00Å (CH), and O—H = 0.87 Å, and with *U*_iso_(H) = 1.2 *U*_eq_(C). H atoms of hydroxy obtained from the difference Fourier synthesized, and amended to the *x*, *y* and *z* coordinates and *U*_eq_ for least-squares. In the absence of significant anomalous scattering effects, Friedel pairs were merged. The choice of enantiomer was based on comparison of the optical rotation with that of related compounds with known stereochemistry.

### Crystal data

**Table:**

C_20_H_30_O_6_	*F*(000) = 792
*M**_r_* = 366.44	*D*_x_ = 1.328 Mg m^−^^3^
Monoclinic, *C*2	Melting point = 531–533 K
Hall symbol: C 2y	Mo *K*α radiation, λ = 0.71073 Å
*a* = 21.581 (11) Å	Cell parameters from 3276 reflections
*b* = 6.111 (3) Å	θ = 3.2–27.5°
*c* = 14.080 (7) Å	µ = 0.10 mm^−^^1^
β = 99.129 (8)°	*T* = 93 K
*V* = 1833.3 (16) Å^3^	Prism, colorless
*Z* = 4	0.60 × 0.18 × 0.14 mm

### Data collection

**Table:**

Rigaku AFC10/Saturn724+ diffractometer	2291 independent reflections
Radiation source: rotating anode	1853 reflections with *I* > 2σ(*I*)
graphite	*R*_int_ = 0.052
Detector resolution: 28.5714 pixels mm^-1^	θ_max_ = 27.5°, θ_min_ = 3.2°
phi and ω scans	*h* = −26→27
Absorption correction: multi-scan (*ABSCOR*; Higashi, 1995)	*k* = −7→7
*T*_min_ = 0.944, *T*_max_ = 0.987	*l* = −18→16
7255 measured reflections	

### Refinement

**Table:**

Refinement on *F*^2^	Primary atom site location: structure-invariant direct methods
Least-squares matrix: full	Secondary atom site location: difference Fourier map
*R*[*F*^2^ > 2σ(*F*^2^)] = 0.039	Hydrogen site location: inferred from neighbouring sites
*wR*(*F*^2^) = 0.079	H atoms treated by a mixture of independent and constrained refinement
*S* = 1.00	*w* = 1/[σ^2^(*F*_o_^2^) + (0.0202*P*)^2^ + 0.356*P*] where *P* = (*F*_o_^2^ + 2*F*_c_^2^)/3
2291 reflections	(Δ/σ)_max_ < 0.001
257 parameters	Δρ_max_ = 0.31 e Å^−^^3^
1 restraint	Δρ_min_ = −0.21 e Å^−^^3^

### Special details

**Table:**

Geometry. All e.s.d.'s (except the e.s.d. in the dihedral angle between two l.s. planes) are estimated using the full covariance matrix. The cell e.s.d.'s are taken into account individually in the estimation of e.s.d.'s in distances, angles and torsion angles; correlations between e.s.d.'s in cell parameters are only used when they are defined by crystal symmetry. An approximate (isotropic) treatment of cell e.s.d.'s is used for estimating e.s.d.'s involving l.s. planes.
Refinement. Refinement of *F*^2^ against ALL reflections. The weighted *R*-factor *wR* and goodness of fit *S* are based on *F*^2^, conventional *R*-factors *R* are based on *F*, with *F* set to zero for negative *F*^2^. The threshold expression of *F*^2^ > 2σ(*F*^2^) is used only for calculating *R*-factors(gt) *etc*. and is not relevant to the choice of reflections for refinement. *R*-factors based on *F*^2^ are statistically about twice as large as those based on *F*, and *R*-factors based on ALL data will be even larger.

### Fractional atomic coordinates and isotropic or equivalent isotropic displacement parameters (Å^2^)

**Table:**

	*x*	*y*	*z*	*U*_iso_*/*U*_eq_	
O1	0.31297 (8)	0.1972 (3)	0.27447 (11)	0.0204 (4)	
O2	0.33579 (8)	0.6630 (3)	0.49510 (11)	0.0188 (4)	
O3	0.32260 (8)	0.5439 (3)	0.06465 (11)	0.0176 (4)	
O4	0.27091 (9)	0.1533 (3)	0.11757 (12)	0.0207 (4)	
O5	0.22162 (10)	0.5024 (3)	0.43939 (12)	0.0225 (5)	
O6	0.21732 (9)	0.7558 (3)	0.08613 (12)	0.0174 (4)	
C1	0.35369 (11)	0.7387 (4)	0.40541 (16)	0.0163 (6)	
H1	0.3340	0.8853	0.3904	0.020*	
C2	0.42453 (11)	0.7690 (5)	0.42134 (17)	0.0206 (6)	
H2A	0.4451	0.6300	0.4447	0.025*	
H2B	0.4364	0.8818	0.4714	0.025*	
C3	0.44778 (12)	0.8383 (5)	0.32867 (17)	0.0213 (6)	
H3A	0.4937	0.8623	0.3424	0.026*	
H3B	0.4277	0.9786	0.3060	0.026*	
C4	0.43308 (12)	0.6660 (5)	0.24870 (17)	0.0195 (6)	
C5	0.36084 (11)	0.6243 (4)	0.23254 (16)	0.0148 (6)	
H5	0.3417	0.7623	0.2031	0.018*	
C6	0.34003 (12)	0.4452 (4)	0.15804 (16)	0.0155 (6)	
H6	0.3764	0.3450	0.1555	0.019*	
C7	0.28650 (12)	0.3120 (4)	0.18823 (16)	0.0163 (6)	
C8	0.23046 (12)	0.4508 (4)	0.20869 (16)	0.0147 (5)	
C9	0.25655 (11)	0.6250 (4)	0.28591 (16)	0.0139 (5)	
H9	0.2554	0.7663	0.2498	0.017*	
C10	0.32775 (11)	0.5824 (4)	0.32303 (16)	0.0140 (5)	
C11	0.21320 (11)	0.6621 (5)	0.36261 (16)	0.0170 (6)	
H11	0.2233	0.8093	0.3921	0.020*	
C12	0.14368 (11)	0.6607 (5)	0.32056 (18)	0.0228 (6)	
H12A	0.1316	0.8079	0.2946	0.027*	
H12B	0.1191	0.6302	0.3727	0.027*	
C13	0.12622 (13)	0.4885 (5)	0.23918 (18)	0.0238 (7)	
H13	0.0845	0.4202	0.2424	0.029*	
C14	0.17780 (12)	0.3148 (5)	0.24270 (18)	0.0215 (6)	
H14A	0.1645	0.1916	0.1985	0.026*	
H14B	0.1907	0.2577	0.3087	0.026*	
C15	0.19233 (12)	0.5566 (5)	0.11644 (17)	0.0181 (6)	
H15	0.1888	0.4485	0.0625	0.022*	
C16	0.12735 (13)	0.5961 (5)	0.14211 (19)	0.0292 (7)	
C17	0.08297 (14)	0.7116 (6)	0.09052 (19)	0.0371 (9)	
H17A	0.0444	0.7361	0.1132	0.045*	
H17B	0.0894	0.7707	0.0305	0.045*	
C18	0.44957 (12)	0.7592 (6)	0.15453 (18)	0.0278 (7)	
H18A	0.4943	0.7971	0.1636	0.033*	
H18B	0.4244	0.8907	0.1365	0.033*	
H18C	0.4406	0.6495	0.1035	0.033*	
C19	0.47522 (13)	0.4647 (5)	0.2747 (2)	0.0282 (7)	
H19A	0.4644	0.3509	0.2258	0.034*	
H19B	0.4689	0.4084	0.3376	0.034*	
H19C	0.5193	0.5065	0.2772	0.034*	
C20	0.33680 (12)	0.3413 (4)	0.35333 (17)	0.0168 (6)	
H20A	0.3820	0.3119	0.3744	0.020*	
H20B	0.3145	0.3122	0.4083	0.020*	
H2O	0.3212 (12)	0.785 (5)	0.5241 (19)	0.029 (8)*	
H3O	0.3098 (15)	0.433 (6)	0.025 (2)	0.054 (12)*	
H4O	0.2434 (12)	0.061 (5)	0.1325 (18)	0.024 (8)*	
H5O	0.2608 (14)	0.529 (5)	0.4670 (18)	0.025 (8)*	
H6O	0.2500 (13)	0.724 (5)	0.0724 (19)	0.027 (9)*	

### Atomic displacement parameters (Å^2^)

**Table:**

	*U*^11^	*U*^22^	*U*^33^	*U*^12^	*U*^13^	*U*^23^
O1	0.0360 (11)	0.0146 (10)	0.0091 (8)	−0.0002 (8)	−0.0015 (7)	0.0003 (8)
O2	0.0292 (11)	0.0195 (10)	0.0080 (8)	0.0025 (9)	0.0046 (7)	0.0004 (8)
O3	0.0243 (10)	0.0194 (11)	0.0089 (9)	−0.0021 (8)	0.0023 (7)	−0.0002 (9)
O4	0.0352 (12)	0.0143 (10)	0.0126 (9)	−0.0068 (9)	0.0038 (8)	−0.0032 (9)
O5	0.0291 (12)	0.0262 (11)	0.0125 (9)	−0.0036 (9)	0.0042 (8)	0.0045 (8)
O6	0.0238 (11)	0.0163 (10)	0.0120 (9)	0.0011 (9)	0.0024 (8)	0.0024 (8)
C1	0.0250 (15)	0.0167 (14)	0.0077 (11)	0.0008 (11)	0.0042 (10)	−0.0002 (11)
C2	0.0247 (15)	0.0240 (15)	0.0119 (12)	−0.0005 (13)	−0.0008 (10)	−0.0062 (12)
C3	0.0185 (14)	0.0249 (16)	0.0205 (14)	−0.0054 (12)	0.0028 (11)	−0.0045 (13)
C4	0.0201 (14)	0.0241 (15)	0.0147 (12)	−0.0021 (12)	0.0045 (10)	−0.0046 (12)
C5	0.0198 (14)	0.0142 (14)	0.0102 (12)	0.0000 (11)	0.0017 (9)	0.0000 (11)
C6	0.0187 (14)	0.0179 (14)	0.0090 (12)	0.0012 (11)	−0.0002 (10)	0.0003 (11)
C7	0.0270 (15)	0.0123 (13)	0.0087 (12)	−0.0012 (11)	0.0000 (10)	−0.0009 (11)
C8	0.0177 (14)	0.0164 (14)	0.0095 (12)	−0.0043 (11)	0.0004 (10)	−0.0002 (11)
C9	0.0191 (13)	0.0132 (13)	0.0101 (12)	−0.0001 (11)	0.0040 (9)	0.0027 (11)
C10	0.0174 (13)	0.0163 (14)	0.0080 (11)	−0.0009 (11)	0.0009 (9)	−0.0018 (11)
C11	0.0235 (14)	0.0175 (13)	0.0104 (12)	0.0009 (12)	0.0041 (9)	0.0007 (12)
C12	0.0180 (14)	0.0295 (16)	0.0219 (14)	−0.0016 (13)	0.0060 (10)	0.0016 (14)
C13	0.0214 (15)	0.0337 (18)	0.0160 (13)	−0.0112 (13)	0.0026 (11)	0.0010 (13)
C14	0.0293 (16)	0.0221 (15)	0.0128 (13)	−0.0099 (13)	0.0028 (11)	−0.0025 (12)
C15	0.0242 (15)	0.0181 (14)	0.0113 (12)	−0.0045 (12)	0.0005 (10)	0.0006 (12)
C16	0.0216 (15)	0.046 (2)	0.0193 (14)	−0.0025 (15)	0.0008 (11)	0.0059 (15)
C17	0.0295 (16)	0.061 (2)	0.0218 (15)	0.0133 (16)	0.0072 (12)	0.0133 (16)
C18	0.0257 (16)	0.0389 (19)	0.0207 (14)	−0.0132 (14)	0.0092 (11)	−0.0052 (14)
C19	0.0194 (15)	0.0378 (18)	0.0268 (15)	0.0035 (14)	0.0020 (12)	−0.0095 (15)
C20	0.0222 (14)	0.0178 (14)	0.0093 (12)	0.0012 (11)	−0.0008 (10)	−0.0019 (11)

### Geometric parameters (Å, °)

**Table:**

O1—C7	1.440 (3)	C8—C14	1.544 (3)
O1—C20	1.446 (3)	C8—C9	1.562 (3)
O2—C1	1.454 (3)	C8—C15	1.563 (3)
O2—H2O	0.93 (3)	C9—C11	1.554 (3)
O3—C6	1.441 (3)	C9—C10	1.564 (3)
O3—H3O	0.89 (3)	C9—H9	1.0000
O4—C7	1.392 (3)	C10—C20	1.538 (4)
O4—H4O	0.87 (3)	C11—C12	1.523 (3)
O5—C11	1.446 (3)	C11—H11	1.0000
O5—H5O	0.89 (3)	C12—C13	1.557 (4)
O6—C15	1.424 (3)	C12—H12A	0.9900
O6—H6O	0.78 (3)	C12—H12B	0.9900
C1—C2	1.521 (3)	C13—C16	1.520 (4)
C1—C10	1.538 (3)	C13—C14	1.533 (4)
C1—H1	1.0000	C13—H13	1.0000
C2—C3	1.530 (3)	C14—H14A	0.9900
C2—H2A	0.9900	C14—H14B	0.9900
C2—H2B	0.9900	C15—C16	1.522 (4)
C3—C4	1.537 (3)	C15—H15	1.0000
C3—H3A	0.9900	C16—C17	1.312 (4)
C3—H3B	0.9900	C17—H17A	0.9500
C4—C18	1.536 (3)	C17—H17B	0.9500
C4—C19	1.539 (4)	C18—H18A	0.9800
C4—C5	1.560 (3)	C18—H18B	0.9800
C5—C6	1.533 (3)	C18—H18C	0.9800
C5—C10	1.577 (3)	C19—H19A	0.9800
C5—H5	1.0000	C19—H19B	0.9800
C6—C7	1.528 (3)	C19—H19C	0.9800
C6—H6	1.0000	C20—H20A	0.9900
C7—C8	1.541 (4)	C20—H20B	0.9900
			
C7—O1—C20	113.34 (18)	C20—C10—C1	111.76 (19)
C1—O2—H2O	106.4 (18)	C20—C10—C9	109.1 (2)
C6—O3—H3O	105 (2)	C1—C10—C9	111.7 (2)
C7—O4—H4O	112.2 (17)	C20—C10—C5	109.0 (2)
C11—O5—H5O	101.6 (18)	C1—C10—C5	110.6 (2)
C15—O6—H6O	105 (2)	C9—C10—C5	104.46 (18)
O2—C1—C2	108.07 (18)	O5—C11—C12	106.6 (2)
O2—C1—C10	110.0 (2)	O5—C11—C9	113.8 (2)
C2—C1—C10	115.1 (2)	C12—C11—C9	113.18 (19)
O2—C1—H1	107.8	O5—C11—H11	107.7
C2—C1—H1	107.8	C12—C11—H11	107.7
C10—C1—H1	107.8	C9—C11—H11	107.7
C1—C2—C3	111.5 (2)	C11—C12—C13	113.5 (2)
C1—C2—H2A	109.3	C11—C12—H12A	108.9
C3—C2—H2A	109.3	C13—C12—H12A	108.9
C1—C2—H2B	109.3	C11—C12—H12B	108.9
C3—C2—H2B	109.3	C13—C12—H12B	108.9
H2A—C2—H2B	108.0	H12A—C12—H12B	107.7
C2—C3—C4	112.2 (2)	C16—C13—C14	102.3 (2)
C2—C3—H3A	109.2	C16—C13—C12	109.4 (2)
C4—C3—H3A	109.2	C14—C13—C12	110.7 (2)
C2—C3—H3B	109.2	C16—C13—H13	111.3
C4—C3—H3B	109.2	C14—C13—H13	111.3
H3A—C3—H3B	107.9	C12—C13—H13	111.3
C18—C4—C3	109.2 (2)	C13—C14—C8	100.6 (2)
C18—C4—C19	107.0 (2)	C13—C14—H14A	111.7
C3—C4—C19	109.1 (2)	C8—C14—H14A	111.7
C18—C4—C5	107.32 (19)	C13—C14—H14B	111.7
C3—C4—C5	107.7 (2)	C8—C14—H14B	111.7
C19—C4—C5	116.3 (2)	H14A—C14—H14B	109.4
C6—C5—C4	113.20 (19)	O6—C15—C16	110.0 (2)
C6—C5—C10	108.40 (19)	O6—C15—C8	115.4 (2)
C4—C5—C10	118.60 (19)	C16—C15—C8	104.6 (2)
C6—C5—H5	105.1	O6—C15—H15	108.9
C4—C5—H5	105.1	C16—C15—H15	108.9
C10—C5—H5	105.1	C8—C15—H15	108.9
O3—C6—C7	112.17 (19)	C17—C16—C13	128.0 (3)
O3—C6—C5	109.4 (2)	C17—C16—C15	125.0 (3)
C7—C6—C5	110.02 (19)	C13—C16—C15	107.0 (2)
O3—C6—H6	108.4	C16—C17—H17A	120.0
C7—C6—H6	108.4	C16—C17—H17B	120.0
C5—C6—H6	108.4	H17A—C17—H17B	120.0
O4—C7—O1	106.4 (2)	C4—C18—H18A	109.5
O4—C7—C6	106.3 (2)	C4—C18—H18B	109.5
O1—C7—C6	106.13 (19)	H18A—C18—H18B	109.5
O4—C7—C8	114.2 (2)	C4—C18—H18C	109.5
O1—C7—C8	109.18 (19)	H18A—C18—H18C	109.5
C6—C7—C8	114.2 (2)	H18B—C18—H18C	109.5
C7—C8—C14	113.6 (2)	C4—C19—H19A	109.5
C7—C8—C9	107.36 (19)	C4—C19—H19B	109.5
C14—C8—C9	110.66 (19)	H19A—C19—H19B	109.5
C7—C8—C15	113.4 (2)	C4—C19—H19C	109.5
C14—C8—C15	99.4 (2)	H19A—C19—H19C	109.5
C9—C8—C15	112.4 (2)	H19B—C19—H19C	109.5
C11—C9—C8	113.1 (2)	O1—C20—C10	110.87 (18)
C11—C9—C10	117.39 (18)	O1—C20—H20A	109.5
C8—C9—C10	110.2 (2)	C10—C20—H20A	109.5
C11—C9—H9	104.9	O1—C20—H20B	109.5
C8—C9—H9	104.9	C10—C20—H20B	109.5
C10—C9—H9	104.9	H20A—C20—H20B	108.1
			
O2—C1—C2—C3	177.3 (2)	C2—C1—C10—C5	−42.7 (3)
C10—C1—C2—C3	53.9 (3)	C11—C9—C10—C20	80.4 (3)
C1—C2—C3—C4	−61.2 (3)	C8—C9—C10—C20	−51.1 (2)
C2—C3—C4—C18	172.4 (2)	C11—C9—C10—C1	−43.6 (3)
C2—C3—C4—C19	−70.9 (3)	C8—C9—C10—C1	−175.10 (19)
C2—C3—C4—C5	56.2 (3)	C11—C9—C10—C5	−163.1 (2)
C18—C4—C5—C6	65.8 (3)	C8—C9—C10—C5	65.4 (2)
C3—C4—C5—C6	−176.8 (2)	C6—C5—C10—C20	49.2 (2)
C19—C4—C5—C6	−54.0 (3)	C4—C5—C10—C20	−81.7 (3)
C18—C4—C5—C10	−165.6 (2)	C6—C5—C10—C1	172.4 (2)
C3—C4—C5—C10	−48.1 (3)	C4—C5—C10—C1	41.5 (3)
C19—C4—C5—C10	74.7 (3)	C6—C5—C10—C9	−67.3 (2)
C4—C5—C6—O3	−92.9 (2)	C4—C5—C10—C9	161.8 (2)
C10—C5—C6—O3	133.3 (2)	C8—C9—C11—O5	82.5 (2)
C4—C5—C6—C7	143.5 (2)	C10—C9—C11—O5	−47.6 (3)
C10—C5—C6—C7	9.7 (3)	C8—C9—C11—C12	−39.4 (3)
C20—O1—C7—O4	174.94 (19)	C10—C9—C11—C12	−169.5 (2)
C20—O1—C7—C6	62.1 (2)	O5—C11—C12—C13	−87.9 (2)
C20—O1—C7—C8	−61.4 (3)	C9—C11—C12—C13	37.9 (3)
O3—C6—C7—O4	58.5 (3)	C11—C12—C13—C16	−93.9 (3)
C5—C6—C7—O4	−179.55 (19)	C11—C12—C13—C14	18.2 (3)
O3—C6—C7—O1	171.43 (19)	C16—C13—C14—C8	45.9 (2)
C5—C6—C7—O1	−66.6 (2)	C12—C13—C14—C8	−70.7 (2)
O3—C6—C7—C8	−68.3 (3)	C7—C8—C14—C13	−170.51 (19)
C5—C6—C7—C8	53.7 (3)	C9—C8—C14—C13	68.7 (2)
O4—C7—C8—C14	59.6 (3)	C15—C8—C14—C13	−49.7 (2)
O1—C7—C8—C14	−59.3 (3)	C7—C8—C15—O6	−82.9 (3)
C6—C7—C8—C14	−177.88 (18)	C14—C8—C15—O6	156.1 (2)
O4—C7—C8—C9	−177.7 (2)	C9—C8—C15—O6	39.0 (3)
O1—C7—C8—C9	63.4 (2)	C7—C8—C15—C16	156.0 (2)
C6—C7—C8—C9	−55.2 (2)	C14—C8—C15—C16	35.1 (3)
O4—C7—C8—C15	−53.0 (3)	C9—C8—C15—C16	−82.0 (3)
O1—C7—C8—C15	−171.9 (2)	C14—C13—C16—C17	159.5 (3)
C6—C7—C8—C15	69.5 (3)	C12—C13—C16—C17	−83.0 (4)
C7—C8—C9—C11	−139.8 (2)	C14—C13—C16—C15	−23.7 (3)
C14—C8—C9—C11	−15.3 (3)	C12—C13—C16—C15	93.8 (3)
C15—C8—C9—C11	94.9 (2)	O6—C15—C16—C17	45.0 (4)
C7—C8—C9—C10	−6.1 (3)	C8—C15—C16—C17	169.5 (3)
C14—C8—C9—C10	118.4 (2)	O6—C15—C16—C13	−131.9 (2)
C15—C8—C9—C10	−131.5 (2)	C8—C15—C16—C13	−7.4 (3)
O2—C1—C10—C20	−43.5 (3)	C7—O1—C20—C10	−0.5 (3)
C2—C1—C10—C20	78.9 (3)	C1—C10—C20—O1	−179.14 (19)
O2—C1—C10—C9	79.1 (2)	C9—C10—C20—O1	56.9 (3)
C2—C1—C10—C9	−158.6 (2)	C5—C10—C20—O1	−56.6 (3)
O2—C1—C10—C5	−165.08 (19)		

### Hydrogen-bond geometry (Å, °)

**Table:**

*D*—H···*A*	*D*—H	H···*A*	*D*···*A*	*D*—H···*A*
O2—H2O···O5^i^	0.93 (3)	1.74 (3)	2.655 (3)	167 (3)
O4—H4O···O6^ii^	0.87 (3)	2.02 (3)	2.696 (3)	133 (2)
O3—H3O···O6^iii^	0.89 (3)	1.92 (3)	2.787 (3)	164 (3)
O5—H5O···O2	0.89 (3)	1.80 (3)	2.652 (3)	160 (3)
O6—H6O···O3	0.78 (3)	1.93 (3)	2.674 (3)	157 (3)

Symmetry codes: (i) −*x*+1/2, *y*+1/2, −*z*+1; (ii) *x*, *y*−1, *z*; (iii) −*x*+1/2, *y*−1/2, −*z*.

## References

[bb1] Allen, F. H., Kennard, O., Watson, D. G., Brammer, L., Orpen, A. G. & Taylor, R. (1987). *J. Chem. Soc. Perkin Trans. 2*, pp. S1–19.

[bb2] Higashi, T. (1995). *ABSCOR* Rigaku Corporation, Tokyo, Japan.

[bb3] Rigaku (2008). *CrystalClear* Rigaku Corporation, The Woodlands, Texas, USA.

[bb4] Sheldrick, G. M. (2008). *Acta Cryst.* A**64**, 112–122. 10.1107/S0108767307043930 18156677

[bb5] Siemens (1995). *SHELXTL* Siemens Industrial Automation Inc., Analytical Instrumentation, Madison, Wisconsin, USA.

[bb6] Sun, H. D., Xu, Y. L. & Jiang, B. (2001). *Diterpenoids from Isodon Species*, pp. 4–17, 239. Beijing: Science Press.

[bb7] Wang, X. R., Hu, H. P., Wang, H. P., Wang, S. Q., Ueda, S. & Fujita, T. (1994). *Phytochemistry*, **37**, 1367–1370.

[bb8] Yan, F. L., Wang, C. M., Guo, L. Q., Zhang, J. X. & Sun, H. D. (2008). *J. Chem. Res.* **9**, 522–524.

